# The Implications of Microglial Regulation in Neuroplasticity-Dependent Stroke Recovery

**DOI:** 10.3390/biom13030571

**Published:** 2023-03-21

**Authors:** Chenye Qiao, Zongjian Liu, Shuyan Qie

**Affiliations:** Department of Rehabilitation, Beijing Rehabilitation Hospital, Capital Medical University, Beijing 100144, China

**Keywords:** stroke, neuroplasticity, microglia, microglial phagocytosis, rehabilitation, neuromodulation

## Abstract

Stroke causes varying degrees of neurological deficits, leading to corresponding dysfunctions. There are different therapeutic principles for each stage of pathological development. Neuroprotection is the main treatment in the acute phase, and functional recovery becomes primary in the subacute and chronic phases. Neuroplasticity is considered the basis of functional restoration and neurological rehabilitation after stroke, including the remodeling of dendrites and dendritic spines, axonal sprouting, myelin regeneration, synapse shaping, and neurogenesis. Spatiotemporal development affects the spontaneous rewiring of neural circuits and brain networks. Microglia are resident immune cells in the brain that contribute to homeostasis under physiological conditions. Microglia are activated immediately after stroke, and phenotypic polarization changes and phagocytic function are crucial for regulating focal and global brain inflammation and neurological recovery. We have previously shown that the development of neuroplasticity is spatiotemporally consistent with microglial activation, suggesting that microglia may have a profound impact on neuroplasticity after stroke and may be a key therapeutic target for post-stroke rehabilitation. In this review, we explore the impact of neuroplasticity on post-stroke restoration as well as the functions and mechanisms of microglial activation, polarization, and phagocytosis. This is followed by a summary of microglia-targeted rehabilitative interventions that influence neuroplasticity and promote stroke recovery.

## 1. Introduction

Stroke is a major cause of death and long-term disability, worldwide. Despite constant incidence and declining mortality rates over the past 20 years, the number of stroke survivors continues to decrease [1,2,3]. They are unable to live independently and are more likely to experience subsequent neurological sequelae [4,5]. Stroke can cause focal and global neurological deficits. Different therapeutic principles are adopted in different periods. In the acute stage of stroke, neuroprotection is the main treatment [6]; reducing cerebral ischemia-reperfusion injury (IRI) is also crucial. In the subacute and chronic stages, functional recovery becomes the primary objective. Neuroplasticity is recognized as the basis of functional restoration and neurological rehabilitation after stroke, including remodeling of dendrites and dendritic spines, axonal sprouting, synapse shaping, and neurogenesis. Spontaneous neuroplasticity begins immediately after stroke, reaches a plateau in three to four weeks, and can be sustained in the chronic phase [7]. Spatiotemporal development profoundly affects the reconstruction of neural circuits and brain networks.

Microglia, the resident immune cells of the central nervous system (CNS), play a key role in brain development, homeostasis maintenance, and the disease response of the CNS through phenotypic polarization, morphological changes, and functional transformation. They participate in a variety of pathophysiological processes in the brain, including the promotion of neuronal survival, induction of programmed cell death, immune monitoring and antigen presentation, inflammation regulation, modulation of synaptic activity, synaptic pruning, remodeling, etc. [8,9,10,11,12].

After stroke, the activation, polarization, and phagocytosis of microglia are crucial for regulating the neuroinflammatory microenvironment and enhancing neuroplasticity. Our previous study presented that the development of neuroplasticity overlaps both temporally and spatially with microglial activation [7], suggesting that microglia may have a profound impact on neuroplasticity following stroke and that they may be key therapeutic targets for stroke rehabilitation. In this review, we explore therapeutic targeting at different stages after stroke and the impact of neuroplasticity during this process. We then discuss the functions and mechanisms of microglial activation, polarization, and phagocytosis under physiological and pathological conditions. Finally, we provide a summary of microglia-targeted therapeutic interventions for promoting stroke recovery.

## 2. Pathophysiology and Therapeutic Target of Stroke Recovery

### 2.1. Pathophysiology of Stroke in Different Phases

Stroke commonly comprises two pathological subtypes. Hemorrhagic stroke accounts for approximately 10–15% of stroke cases. During this process, stress in the brain and internal injury cause the rupture of blood vessel [13]. Hematomas compressing brain tissue form for blood leakage into the brain parenchyma. The mass effect of the hematoma combined with neurotoxic effects further causes increased intracranial pressure, cerebral herniation, or death [14,15].

Ischemic stroke is caused by abrupt occlusion of the cerebral artery. The consequent interruption of blood flow and obstruction of the supply of oxygen lead to glutamate excitotoxicity, calcium overload, oxidative and nitrosative stress, and the release of inflammatory mediators, thereby activating a series of detrimental signaling cascades that induce neuronal injury or death [1,2,16,17]. Reversible neuronal impairment occurs after an ischemic attack, leading not only to relevant symptoms but also functional deficits corresponding to the location of the ischemia [18]. The progression of brain damage involves irreversibly injured necrotic tissue in the ischemic core, followed by injury development in the penumbral area, and then expanding to the entire ischemic territory [1,19]. Due to focal and global brain neurological damage following stroke, patients have different degrees of neurological deficits after stroke, such as dyskinesia, sensory dysfunction, swallowing dysfunction, dysarthria, aphasia, cognitive impairment, impaired cardiopulmonary function, mental disorders, and many complications, which further leads to a decline in quality of life and social participation [3,20].

Aside from revascularization therapy(thrombolysis and thrombectomy) and neuroprotective therapies (non-pharmaceutical and pharmaceutical therapies) for managing stroke in different phases [21], rehabilitative therapy helps to alleviate disability by promoting the recovery of impairment, activity, or participation after stroke [22] and is formally associated with a “time frame”, which coincides with the development of stroke and the period of maximal spontaneous recovery [23]. Thus, although rehabilitation plays a key role after stroke, not all stages are suitable for rehabilitative interventions [24]. According to both animal models and human trials, intensive rehabilitation within 24 h is potentially harmful [23]. In a clinical trial, a four-week intervention of physical fitness training did not result in an improvement in activities during the subacute period (days 5–45 after stroke) [25].

The therapeutic targets of stroke recovery vary according to the developmental pathophysiological process (Table 1). In the acute phase (minutes to days), a series of detrimental events occur after acute ischemic injury, including infiltration of peripheral immune cells, activation of resident glial cells, disturbance of ionic homeostasis, oxidative stress, mitochondrial dysfunction, and DNA damage. These processes involve cell necrosis within the lesion core and peri-infarct area. Therapeutic strategies have focused on neuroprotection to prevent neuronal injury and death, reduce infarct volume, and limit the decrease in neuronal density in the penumbra [16,26,27,28,29,30,31]. In addition, reducing IRI is critical. During the restoration of blood perfusion, IRI can lead to cerebral edema and even hemorrhage, thereby exacerbating the detrimental biological cascade response and causing irreversible tissue damage [21,32]. Therefore, besides neuroprotection, effective reduction of IRI is also a key target in the treatment of the acute phase of ischemic stroke [33].

In the subacute phase (days to weeks), the mechanisms are more complicated than in the acute phase and include amplification of local and systemic immune responses, increased cytokine and reactive oxygen species (ROS) production, cell edema, and ion imbalances [28,34]. The activation of several protective mechanisms triggers beneficial repair processes, including neurogenesis and angiogenesis [27]. In addition, many endogenous processes are active, including axonal sprouting, dendrite remodeling, increased levels of growth factors, and altered synaptic and cortical excitability. Some of these processes have been demonstrated to mediate behavioral changes [35].

In the chronic phase (weeks to months), the end of spontaneous structural recovery is marked by stabilization of the post-stroke neurological deficits [35]. The therapeutic priorities should shift from neuroprotection to functional rehabilitation. Post-ischemic inflammatory responses appear to exacerbate tissue damage at an early stage, whereas they are assumed to promote tissue repair and functional restoration during the chronic phase [36]. During this stage, excitotoxicity decreases and the brain milieu becomes primarily inhibitory, and neural repair and excitability enhancement come to the forefront of post-stroke intervention [22,35].

### 2.2. The Basis of Functional Recovery: Neuroplasticity

**Figure 1:**
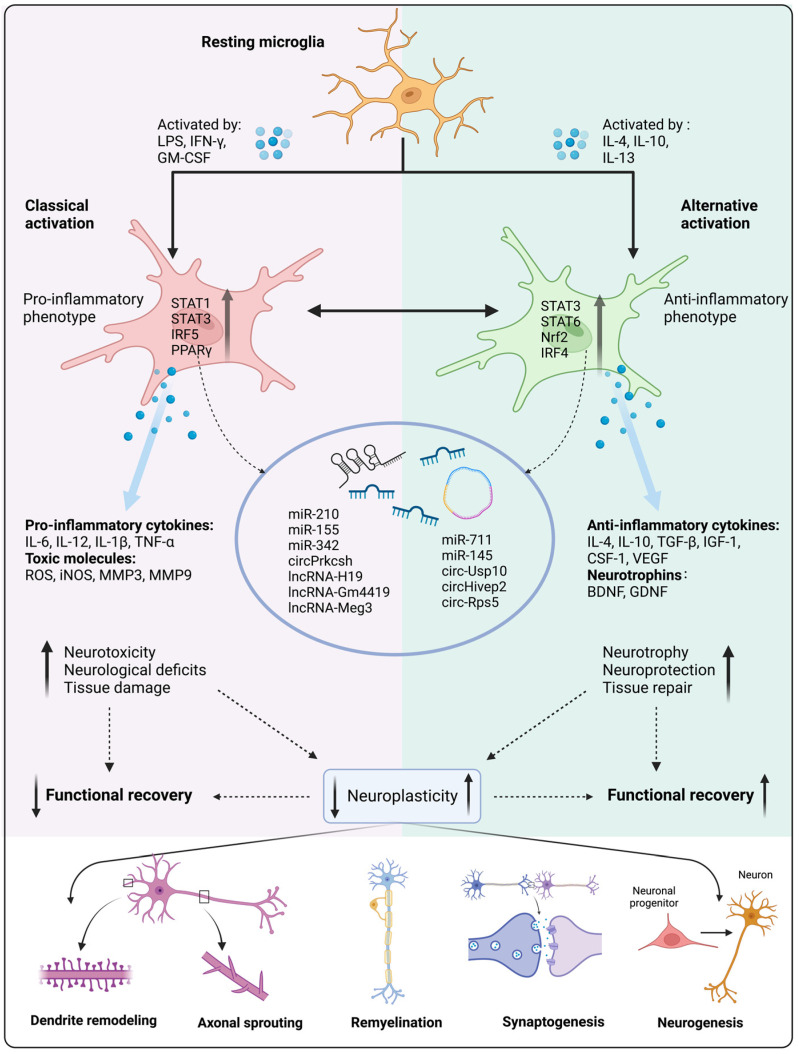
Microglial activation and polarization affect neuroplasticity and functional recovery. Under pathological conditions, microglia can be activated by specific cytokines that upregulate transcription factors of downstream signaling pathways, thereby resulting in pro-inflammatory and anti-inflammatory phenotypic polarization. Non-coding RNA (ncRNA) expression also contributes to direct microglial polarization. Pro-inflammatory microglia tend to release pro-inflammatory factors and toxic molecules, which leads to increased neurotoxicity, neurological deficits, and tissue damage, ultimately leading to attenuated neuroplasticity and functional recovery. Conversely, anti-inflammatory microglia release anti-inflammatory factors and neurotrophins, resulting in increased neurotrophy and neuroprotection, thereby enhancing neuroplasticity and functional recovery. LPS, lipopolysaccharide; IFN-γ, interferon-γ; GM-CSF, granulocyte-macrophage colony-stimulating factor; IL-4, interleukin-4; IL-10, interleukin-10; IL-13, interleukin-13; STAT1, signal transducer and activator of transcription 1; STAT3, signal transducer and activator of transcription 3; IRF5, interferon regulatory factor 5; PPARγ, peroxisome proliferator-activated receptor γ; STAT6, signal transducer and activator of transcription 6; Nrf2, nuclear factor erythroid 2-related factor 2 ; IRF4, interferon regulatory factor 4; IL-6, interleukin-6; IL-12, interleukin-12; IL-1β, interleukin-1β; TNF-α, tumor necrosis factor-α; ROS, reactive oxygen species; iNOS, inducible nitric oxide synthase; MMP3, matrix metalloproteinase 3; MMP9, matrix metalloproteinase 9; TGF-β, transforming growth factor-β; IGF-1, insulin-like growth factor-1; CSF-1, colony-stimulating factor-1; VEGF, vascular endothelial growth factor; BDNF, brain-derived neurotrophic factor; GDNF, glial cell line-derived neurotrophic factor; Meg3, maternally expressed gene 3; Usp10, ubiquitin specific peptidase 10. Created with BioRender.com (accessed on 1 March 2023.

Neuroplasticity underlies the intrinsic reorganization of brain structure and function during the entire lifespan of an individual. Stroke injury causes significant alterations in the neural network within the affected area [37]. Neuroplasticity contributes to spontaneous brain rewiring during post-stroke recovery. This mechanism is closely related to the remodeling of dendrites and dendritic spines, axonal sprouting, synapsis shaping, and synaptogenesis (Figure 1).

#### 2.2.1. Remodeling of Dendrites and Dendritic Spines

The brain must undergo significant modifications to adapt to new challenges caused by the damage after stroke; a key mechanism is the rearrangement of existing dendritic arbors that are associated with new synaptic contacts [38]. Since increased dendritic branching can provide more surface for synaptogenesis and re-establish axonal-dendritic connections, its increase is a key component of neuroplasticity [39]. Dendritic spines, the protrusions on dendrites, are the termination of most excitatory synapses and are relatively stable entities in the brain [40,41,42], but they can undergo changes in the brain microenvironment. Following stroke, a series of structural changes occur around peri-infarct regions to compensate for the injury caused by stroke, including the organization of dendrites and an increase in dendritic spine turnover and spine density. Dendritic spines may be malleable, which makes them a crucial substrate for neuroplasticity that underpins brain remodeling and damage recovery [43,44,45]. Regarding quantity, in the infarct areas, spine density has been reported to return to normal levels, while in areas far away from the infarct with preserved blood flow, spine density was restored faster by adding more spines, eventually surpassing the baseline by 15% [46]. The integrity of the brain network is highly dependent on focal blood flow. Dendrites and spines, as energy-consuming structures of neurons, exhibit the most vulnerable traits following blood supply disruption. Within minutes, dendrites become beaded and spines become distorted; however, these do not represent a loss of synapses, and most dendritic spines still have synaptic contacts. In an animal model, when occluded cerebral vessels are perfused within 20 min, injured dendrites and most spines can restore rapidly [44]. In addition, rehabilitative training plays a key role in dendrites and dendritic spine remodeling; for example, brain stimulation has been reported to optimize synaptic reorganization and facilitate functional recovery after stroke [38].

#### 2.2.2. Axonal Sprouting

As postsynaptic partners with dendritic spines, axonal sprouting has been suggested to take place [47]. After stroke, axons are damaged mainly by disruption of the connection pathways that emanate from the cortex. In a rat model, loss of skilled food retrieval occurred following damage to either the dorsal corticospinal tract (comprising approximately 95% of descending axons) or the ventral corticospinal tract (comprising only approximately 2% of the axons), but it could recover within four weeks [48]. In addition, in spontaneous recovery after stroke, new connections are induced both in the ipsilateral and contralateral hemispheres to the lesion. Axonal sprouting initiates in peri-infarct within the first week, which can be clearly detected through anatomical mapping of cortical circuits one month after stroke [49,50,51] as well as several months after stroke [52]. Contralateral axonal sprouting has been reported in other cortical lesions, including contralateral projections to the striatum, brainstem, and spinal cord [53,54]. Axonal sprouting can be divided into three forms: stroke-reactive, reparative, and bounded axonal sprouting. Reactive axonal sprouting, which is related to tissue repair and scar formation, occurs around and contralateral to the lesion after stroke. If this ability is limited, reparative axonal sprouting can be enhanced by stimulating neuronal growth or by blocking glial growth inhibitors. These are longer axonal protrusions that connect functionally relevant brain regions. On this basis, through manipulating rehabilitative activity such as constraint-induced movement therapy (CIMT) or skilled reaching training, these connections can extend into largely different functional areas. This process is called unbounded axonal sprouting [55]. According to Wahl et al., sequential axonal therapy and rehabilitation training resulted in functional recovery [56] therefore, the principle of axonal protrusion can be used as a therapeutic target for neurorehabilitation after stroke, but the timing is also important. It should be noted that early (within the first week of stroke) delivery of increased behavioral training and axonal sprouting therapy leads to behavioral deterioration [55].

#### 2.2.3. Myelin Regeneration

Cerebral ischemic injury also leads to demyelination, also known as disruption of the myelin sheath, leading to oligodendrocyte (OL) damage [57] and myelin loss [58], resulting in functional deficits after stroke [59], such as long-term sensorimotor and cognitive impairment [60]. Myelin regeneration begins when oligodendrocyte precursor cells (OPCs) change from a quiescent state to a regenerative phenotype in response to injury around the lesion area [61]. OPCs accumulate and expand within injured areas through proliferation and migration. They then undergo differentiation to promote the growth of new myelin sheaths [62]. Microglia are activated and can remove myelin debris following myelin loss and neuronal injury. This process sets the stage for OPC recruitment and differentiation. In addition, microglia can release soluble factors that play beneficial roles in myelin regeneration and rehabilitation of demyelinating diseases [62,63,64]. Remyelination limits axonal degeneration caused by demyelination and has a neuroprotective effect; therefore, promoting remyelination after cerebral ischemia may be a promising therapeutic strategy to improve functional restoration [65].

#### 2.2.4. Neurogenesis

Neurogenesis comprises endogenous and exogenous regeneration.

Endogenous neurogenesis is closely related to synaptic formation or synaptogenesis, which involves the formation of neurotransmitter-secreted sites in the presynaptic neuron, a receptive field at the postsynaptic partners, and the exact alignment of pre- and post-synapses [66,67]. New synapses are formed throughout the life of an organism and they are especially prominent in the early development of the nervous system [68]. Synapse formation is fundamental for precise wiring of the CNS, which also takes place following stroke. After cerebral ischemic injury in a rodent model, surviving neurons in peri-infarct sites have been shown to undergo spontaneous synaptic remodeling [69]. Growth-associated protein (GAP)-43 and synaptophysin are two proteins involved in neurite outgrowth. GAP-43 is a marker of axon sprouting, and synaptophysin can be used to assess changes in synaptic terminal size or the number of infarctions. An animal study found that after neocortical infarction, axonal sprouting and synaptogenesis occurred in both the peri-infarct and contralateral cortices by observing increased GAP-43 and synaptophysin immunoreactivity [39,70].

Exogenous neurogenesis involves the proliferation and differentiation of progenitor and neural stem cells, as well as the migration and maturation of neuroblasts. In rodent models of stroke, neurogenesis increases after local cerebral ischemia in the ipsilateral subventricular zone (SVZ). Neuroblasts tend to migrate from the SVZ to the ischemic lesion border, where they acquire the characteristics of mature neurons and integrate into local neuronal circuits. This has also been shown to take place in the adult human brain [70,71]. In terms of application, in preclinical studies, neural progenitor cells (NPCs) transplants have shown efficacy in stroke treatment, leading to reduced glial scarring, a lower degree of injury, and better functional performance [72,73]. In many clinical trials, neural stem cell (NSC) replacement has resulted in positive therapeutic effects [74].

## 3. Activation and Polarization of Microglia following Stroke

### 3.1. Microglia in CNS

Neuroplasticity is crucial for the recovery of stroke patients. During this process, as resident immune cells in the CNS, microglia are the first cells to be activated [75,76]. Originating from primitive myeloid progenitor cells in the yolk sac of the embryo, microglia start to appear in the brain on day E9.5. They proliferate via local cell division and are not affected by the hematopoietic system [77,78].

During brain development, microglia can trigger formulated pruning of neurons and selective elimination of deficient synapses, which facilitates the construction of functionally mature neural circuits [79]. This is closely related to the acquisition of learning ability and memory during human growth and development [80].

Under normal physiological conditions, resting microglia exhibit a branched morphology and high motility, which are beneficial for immune monitoring and maintenance of homeostasis. Their branched processes constantly scan the surrounding extracellular space, and their large surface area is conducive to direct communication with neurons, vascular endothelial cells, and other glial cells [76,81,82]. In addition, microglia exhibit high levels of plasticity and adaptability to the environment [82]. In pathological states of the brain, such as stroke, brain tumors, degenerative diseases, and brain injury, microglia detect antigens through pattern recognition receptors. With ameboid morphological changes, activated microglia proliferate and migrate in a tropistic manner [83,84]. Microglia then damage or repair the brain tissue and regulate the microenvironment of the CNS by participating in the secretion of inflammatory cytokines, complement, free radicals, trophic factors, etc. [85].

Under pathological conditions, resting microglia are activated and can be polarized into two extreme states: M1 and M2 [85] (Figure 1). M1 activation, known as the classically activated phenotype, is induced by lipopolysaccharide (LPS), granulocyte-macrophage colony-stimulating factor (GM-CSF), and interferon-γ (IFN-γ), and is thought to be a pro-inflammatory and neurotoxic state. It is characterized by the secretion of pro-inflammatory cytokines and chemokines such as interleukin (IL)-1β, IL-6, IL-12, and tumor necrosis factor-α (TNF-α) as well as the production of neurotoxic substances, including ROS, inducible nitric oxide synthase (iNOS), and proteolytic enzymes including matrix metalloproteinase (MMP)3 and MMP9 [9,86,87,88]. Double-negative T cells (DNTs) have been found to secrete large amounts of TNF-α, promoting the activation of NOD-like receptor thermal protein domain associated protein 3 (NLRP3) and inducing microglia pro-inflammatory polarization [89]. DNT-intrinsic Fas ligand (FasL) and protein tyrosine phosphatase non-receptor type 2 (PTPN2) can collaboratively regulate microglial polarization via TNF-α production. FasL enhances TNF-α production and promotes microglia-mediated neuroinflammation and cerebral ischemic injury, whereas PTPN2 negatively regulates TNF-α production [90].

M2 activation, also known as alternatively activated, is induced by IL-4, IL-13, and IL-10 to secrete anti-inflammatory cytokines, such as IL-10 and transforming growth factor-β (TGF-β). They also release growth factors such as insulin-like growth factor-1 (IGF-1), colony-stimulating factor-1 (CSF-1), vascular endothelial growth factor (VEGF), and neurotrophins such as brain-derived neurotrophic factor (BDNF) and glial cell line-derived neurotrophic factor (GDNF), and their markers such as arginase-1 (Arg-1), chitinase-like protein 3 (also known as Ym1), cluster of differentiation (CD)163, and CD206, which are involved in tissue regeneration and repair, removal of cell debris, provision of trophic factors, and preservation of tissue dynamics following infection or damage [9,75,86,87,91,92]. The M2 phenotype can be further divided into three subclasses: M2a, M2b, and M2c [93]. M2a microglia can prevent inflammation and promote tissue repair and regeneration [94], M2b microglia are considered an intermediate state of microglia with an immunomodulatory phenotype [95], and M2c microglia are involved in tissue remodeling after inflammation resolves [96]. Indeed, microglia can rapidly migrate between states and express both M1- and M2-like markers [97]. The classification of M1 and M2 phenotypes is clear under in vitro conditions, but it is unclear how this classification applies in vivo. Additionally, many researchers have proposed that the use of M1 and M2 to describe the polarization phenotype of microglia is not rigorous and lacks accuracy [82,98].

The common phenotypes of microglia include pro-inflammatory and anti-inflammatory types; however, single-cell sequencing can successfully identify various specific microglial subsets at different developmental stages and pathological states. As an effective and unbiased high-throughput detection technique, single-cell sequencing greatly promotes the understanding of microglial heterogeneity [99,100]. An animal study demonstrated large differences in microglial gene expression that maintain CNS homeostasis during embryonic and postnatal development [101]. Two new subtypes have been identified in the brains of aged mice [102]. In a disease state, in a mouse model of Alzheimer’s disease (AD), the microglial subtype was found to be associated with neurodegenerative diseases (DAM) [103]. In human brain tissue sections of idiopathic Parkinson’s disease (PD), a special microglial cluster involving an inflammatory response has been identified [104] (Table 2). While single-cell sequencing has a great impact on the classification and exploration of microglia subpopulations, how to regulate and maintain microglia in a beneficial state remains to be elucidated by further studies.

### 3.2. Spatiotemporal Distribution of Microglia and Colocalization between Microglia Activation and Neuroplasticity after Stroke

Stroke can trigger a strong inflammatory response, leading to neuronal damage or death in the infarct area, thereby destroying the connection between neural circuits and the integrity of brain networks and leading to more extensive structural damage and further corresponding dysfunction. Neuroplasticity, however, can promote the post-stroke recovery. As mentioned above, neuroplasticity begins immediately after brain injury and primarily occurs in the injured core area. Over time, it gradually appears away from the lesion and the contralateral brain. As the basis of neurological rehabilitation after stroke, the spatiotemporal development of neuroplasticity affects the reconstruction of neural circuits and brain network systems. In addition, following stroke, microglia respond to damage-associated molecular patterns (DAMPs) released by injured cells and participate in the immune response together with other glial cells, blood-derived immune cells, and endothelial cells. In the acute stage of brain ischemia, microglia mainly appear in the infarcted areas. They then gradually spread to the surrounding and remote areas, continuing to the chronic phase after stroke, which is closely related to functional recovery [105]. According to clinical studies, microglial activation can be detected in the acute, subacute, and chronic stages of ischemic stroke [93]. Experimental animal models of ischemic stroke have shown that state changes of microglial activation at different phases depend on the severity of the ischemia [87,106]. Thus, the occurrence of neuroplasticity after stroke is relatively consistent with the distribution and development of microglial activation in time and space, which was also mentioned in our previous review, called spatiotemporal colocalization [7]. The precise temporal and spatial changes in microglia after stroke and the corresponding morphological changes are summarized in detail below.

In the acute and subacute phases, as early as 30 min after the onset of middle cerebral artery occlusion (MCAO), activated microglia could be detected in the periphery of the ischemic lesion [107]. Other animal studies have shown that microglia activated after ischemia are present in the infarct core and border zones at 24 h, continuously increase up to 72 h, and persist for weeks after the initial injury [108]. Both in vitro and in vivo, M2-like microglia appear primarily at the site of injury. They have been reported to be detectable within 12 h and to transiently increase 1–3 days after ischemia. From day 5 to day 7 after injury, microglia mainly expressed the M1 gene and continued to increase within 14 days after injury [75]. During the chronic phase, activated microglia are mostly located in the peri-infarct and distal regions of the human brain. Sustained activation can be maintained for weeks or even years [93].

The number of activated microglia in mouse brain has been reported to peak at approximately one week after ischemia and to decrease at approximately one month after ischemia. In animal models, activated microglia distant from the infarct can be observed over time, which is also thought to be associated with delayed progressive neurodegenerative diseases [109]. Two weeks after injury, microglia were present in the thalamus ipsilateral to the infarct zone and in the distal brainstem corticospinal tract [110]. Six months after stroke, microglial activation declined in the peri-infarct region but remained in remote areas along the corticospinal tract [111].

Although the mechanism of injury after hemorrhagic stroke is different, the inflammatory trigger mechanisms are similar. There have, however, been few studies on microglial polarization. In mice with intracerebral hemorrhage (ICH), M1-like microglia increased dramatically as early as 6 h after intracerebral hemorrhage and underwent a gradual decline within 14 days. M2-like microglia started to increase on the first day, and they exhibited a rising trend during the first 14 days. The shift from the M1 to the M2 phenotype occurs during the first seven days after hemorrhage [15,112].

It is currently thought that the four morphological changes in microglia following stroke are branching, intermediate, ameboid, and globular states [113]. In the adult rat subventricular zone, farther away from the ischemic lesion, the branches were longer and thinner and the cell soma was smaller, which were considered resting microglia. At the border of the ischemic lesion, the cells exhibited an ameboid morphology, with a larger cell soma and shorter protrusion, and intermediate microglia. In the ischemic core area, the microglia exhibited globular cell soma and thick branches, which are highly active, and their morphology is related to the phagocytic function of microglia [113,114]. It has been suggested in an animal study that microglial deramification occurs in the ischemic core due to less capillary blood flow. When blood flow is blocked, a complete loss of blood flow entirely stalls all microglia processes without structural changes. Thus, in the center of the ischemic focus, activated microglia may also were less, and demonstrated a discontinuous and destructive appearance presenting dystrophic morphology, with small cell bodies and only a few long branches [115,116]. These differences in the morphology and number of microglia should be related to the time of injury development and the accumulation of neutrophils first in perivascular spaces and later in the parenchyma [115].

In summary, microglia exhibit distinct activation profiles depending on the different brain microenvironments and undergo temporal and spatial adaptations to switch their phenotypes depending on the site and severity of the brain injury [7]. As immune cells in the CNS, microglial activation is involved in various aspects of neuroplasticity, such as neuronal connection, axonogenesis, dendritic spine reorganization, and endogenous neurogenesis [9,117]. It plays a key role in regulating local and global brain inflammation and in promoting neurological recovery. After stroke, the development of neuroinflammation, the occurrence of neuroplasticity, and the activation of microglia overlap spatially and temporally. It can be seen that the activation of microglia is consistent with the neuroplasticity after stroke and plays a crucial role in the regulation of the inflammatory response and the recovery of brain function.

### 3.3. Molecular Mechanism of Microglial Activation and Polarization Following Stroke

Microglial activation and polarization are crucial in the pathogenesis of ischemic stroke. Therefore, understanding the molecular switch of microglial activation provides a targeted basis for post-stroke treatment. The key factors of microglial activation are discussed according to the following three aspects: surface receptors, transcription factors, and ncRNAs.

#### 3.3.1. Surface Receptor

**Figure 2:**
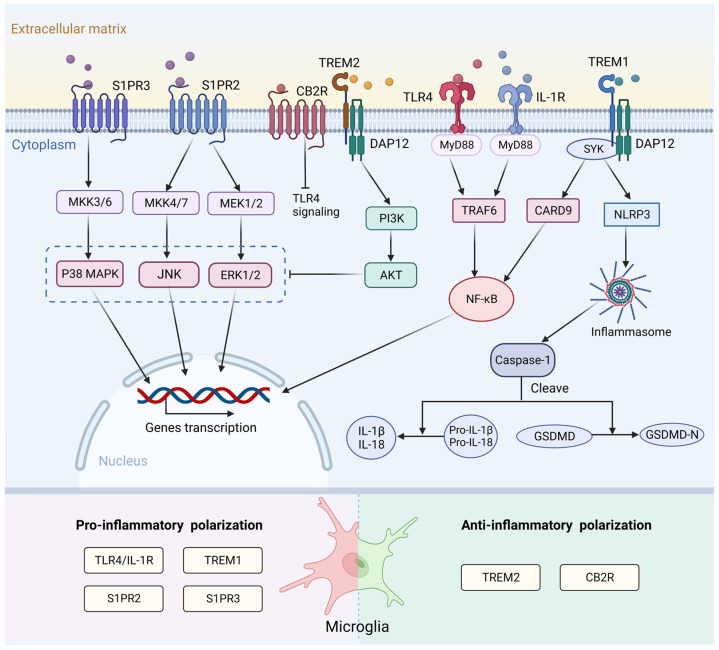
Surface receptors and the signaling pathways associated with microglia activation following stroke. After binding to endogenous ligand, TLR4 and IL-1R activate the MyD88/ NF-κB pathway to increase the release of pro-inflammatory cytokines and induce a pro-inflammatory response. S1PR2 and S1PR3 affect microglial M1 polarization through the ERK1/2 and JNK pathways and p38 MAPK pathway, respectively. TREM1 can activate the downstream CARD9/NF-κB and NLRP3/Caspase-1 signaling pathway to promote the release of inflammatory factors. TREM2- DAP12 interaction activates the PI3K/AKT signaling pathway, which can inhibit TLR4 signaling by blocking MAPK cascade. CB2R exerts anti-inflammatory effects after stroke. This mechanism may be related to inhibition of the TLR4/NF-κB signaling pathway. TLR4, toll-like receptor 4; IL-1R, interleukin-1 receptor; MyD88, myeloid differentiation factor 88; NF-κB, nuclear factor kappa-B; S1PR2, sphingosine-1-phosphate receptor 2; S1PR3, sphingosine-1-phosphate receptor 3; ERK1/2, extracellular signal-regulated kinases 1/2; JNK, c-Jun N-terminal kinase; p38 MAPK, p38 mitogen-activated protein kinase; TREM1, triggering receptor expressed on myeloid cells 1; CARD9, caspase recruitment domain family member 9; NLRP3, NOD-like receptor thermal protein domain associated protein 3; TREM2, triggering receptor expressed on myeloid cells 2; DAP12, DNAX activation protein 12; PI3K, phosphatidylinositol 3-kinase; AKT, protein kinase B; MAPK, mitogen-activated protein kinase; MKK3/6, mitogen-activated protein kinase kinase 3/6; MKK4/7, mitogen-activated protein kinase kinase 4/7; MEK1/2, mitogen-activated protein kinase kinase 1/2; SYK, spleen tyrosine kinase; TRAF6, tumor necrosis factor receptor-associated factor 6; IL-1β, interleukin-1β; IL-18, interleukin-18; GSDMD, Gasdermin D. Created with BioRender.com.

Toll-like receptor 4 (TLR4) is a key regulator of the inflammatory response and is mainly expressed by microglia [118]. After binding to endogenous ligand, it can induce microglial activation and initiate the signaling cascade of the immune response regulated by microglia. This cascade activates Toll/IL-1 receptor (TIR) domain-containing adaptor proteins such as myeloid differentiation factor 88 (MyD88), IκB kinase (IKK), and nuclear factor kappa-B (NF-κB)-induced kinase (NIK). Activated NF-κB then translocates into the nucleus and induces the release of pro-inflammatory cytokines, such as IL-1, IL-6, IL-12, and TNF-α [119,120] (Figure 2). Studies have reported that stimulation of TLR4 can induce activation of hypothalamic microglia in vitro [121]. Increased TLR4 expression is associated with a poor prognosis in mice with ischemic stroke [122]. According to a study using a rat model, inhibition of the TLR4/NF-κB pathway or promotion of TLR4 degradation could prevent microglia-induced neuroinflammation and alleviate post-ischemic stroke or reperfusion injury [123].

Sphingosine-1-phosphate (S1P) is an important bioactive lysophosphatidylcholine. It regulates various biological functions through its five specific G protein-coupled receptors (S1PR1-5) [124,125], which are broadly expressed in microglia [126]. S1P transporters are involved in pro-inflammatory microglial activation [127]. In an ischemia rat model, the addition of S1P to primary microglia increased the level of cytokine IL-17 [128]. S1P receptor 2 (S1PR2) affects M1 polarization through extracellular signal-regulated kinases (ERK)1/2 and c-Jun N-terminal kinase (JNK) pathways in ischemic stroke. Inhibition of S1PR2 can attenuate microglial M1 polarization in post-ischemic brain after transient MCAO (tMCAO) challenge [129]. S1PR3 can also promote pro-inflammatory M1 polarization and activate the p38 mitogen-activated protein kinase (p38 MAPK) pathway after ischemic stroke (Figure 2). In addition, the levels of S1PR3 and its ligand S1P were significantly increased after ICH. Administration of the S1PR3 antagonist CAY10444 to rats attenuated microglia M1 polarization, improved blood-brain barrier (BBB) integrity, and ameliorated behavioral deficits [130].

TREM1 and TREM2 are members of the triggering receptors expressed on myeloid cells (TREM) family. Both receptor types are present in microglia. TREM1 amplifies innate immune responses and is an important inflammatory regulator [120,131]. TREM1 can activate the downstream caspase recruitment domain family member 9 (CARD9)/NF-κB and NLRP3/Caspase-1 signaling pathways both in vitro and in vivo by interacting with spleen tyrosine kinase (SYK) to promote the release of inflammatory factors. Blocking TREM1 can attenuate M1 polarization of microglia and the recruitment of neutrophils, thereby improving the outcome of stroke [131]. TREM2 maintains immune homeostasis and induces anti-inflammatory responses after stroke [132]. TREM2-DNAX activation protein 12 (DAP12) interaction activates the phosphatidylinositol 3-kinase (PI3K)/protein kinase B (AKT) signaling pathway, which subsequently blocks the MAPK cascade and finally inhibits the TLR4-driven microglial inflammatory response [133,134,135] (Figure 2). TREM2 also mediates microglial phagocytosis of apoptotic neurons and cellular debris in various neurological diseases. In a mouse model, TREM2 knockdown in microglia resulted in the ameboid phenotype and reduced phagocytosis of injured neurons [136].

The endocannabinoid (eCB) system, which consists of cannabinoid receptors, ligands, and their metabolic or biosynthetic enzymes, has been reported to trigger anti-inflammatory signaling pathways that regulate immune function [137]. Cannabinoid receptor 2 (CB2R) is significantly expressed in microglia and is closely related to microglial activation and cell migration in resting microglia [138]. Treatment with the CB2R agonist JWH133 decreased the expression of both pro- and anti-inflammatory mediators and led to a lower abundance of Iba1+ microglia. This result indicates a neuroprotective effect and improvement in functional outcomes [139,140,141]. This mechanism may be associated with TLR4/MyD88/NF-κB signaling (Figure 2), but it requires further exploration.

#### 3.3.2. Transcription Factor

The signal transducer and activator of transcription (STAT) family contribute to cytokine release and immune regulation [142]. STAT1 can be activated by Janus kinases (JAKs) and receptor-associated tyrosine kinase proteins [143]. STAT1 is a key transcription factor that modulates the microglial phenotypic transition. In the inflammatory response, after the binding of cytokines to their cognate receptors, JAKs trigger phosphorylation of STAT1, which can increase M1 marker expression in microglia [144]. STAT3 has been implicated in the bidirectional role in regulating microglial polarization [145]. An animal study found that inhibition of JAK2/STAT3 signaling in ischemic stroke promotes the transition of resting microglia to an M2 phenotype and exert a protective role [146]. In addition, a study suggested that activated STAT3 can inhibit the M1 phenotype and promote the M2 phenotype both in vitro and in vivo [147]. STAT6 signaling induces an anti-inflammatory phenotype in microglia. In stroke mice, STAT6 depletion resulted in an increased microglial inflammatory gene signature, reduced clearance of dead and dying neurons, and increased infarction volume [148] (Figure 1).

Nuclear factor erythroid 2-related factor 2 (Nrf2) is known to maintain redox homeostasis and regulate anti-inflammatory signaling pathways (Figure 1). Nrf2 and its related signaling pathways have been shown to play a protective role after stroke [149]. In response to a stimulus, activated Nrf2 moves into the nucleus to enhance the transcription of genes involved in the antioxidant response, including NAD(P)H: quinone oxidoreductase 1 (NQO1), heme oxygenase-1 (HO-1), and other antioxidant proteins [150]. Some studies have shown that in BV2 cells, tanshinol borneol ester (DBZ) exerts antioxidant activity by increasing the transcriptional activity of Nrf2, thereby enhancing HO-1 and NQO1 expression and inhibiting ROS production [151]. Nrf2 is a key regulator of microglial activation during brain inflammation. Nrf2 competes with NF-κB p65 for its common transcriptional co-activator p300/CREB-binding protein (CBP), which counteracts NF-κB-driven inflammation in many animal models [152].

In mammals, interferon regulatory factors (IRFs) comprise nine family members, and the C-terminal variable domain determines the functional specificity of each member [120]. The expression of IRF5 and IRF4 in microglia exhibited a “seesaw” pattern [153] (Figure 1). Downregulation of IRF5 signaling by interfering RNA (siRNA) in cultured primary microglia or conditional knockout (CKO) in a mouse model caused increased IRF4 expression and reduced pro-inflammatory responses, thereby leading to enhanced M2 activation and improved functional recovery, whereas downregulation of IRF4 resulted in increased IRF5 expression, enhanced pro-inflammatory responses, and worse stroke outcomes [154,155]. IRF8 has also been identified as a key transcriptional regulator that transforms microglia into a reactive phenotype [156]. Microglia in IRF8-deficient mice exhibited reduced morphological complexity, Iba1 expression, proliferation, and phagocytosis [157].

Peroxisome proliferator-activated receptor γ (PPARγ) is a ligand-activated transcription factor thought to be a major mediator of inflammatory responses [158] (Figure 1). PPARγ induces the release of TNF-α, IL-1β, intercellular adhesion molecule-1 (ICAM-1), and vascular cell adhesion molecules, which drive the aggregation of macrophages and activation of microglia in the ischemic region [159]. The PPARγ antagonist T0070907 enhanced the expression of M2 markers and decreased the expression of M1 markers in BV2 microglial cells, thus inhibiting NF-κB-IKKβ activation. This suggests that PPARγ inhibition triggers anti-inflammatory responses and alters microglial polarization to the M2 phenotype [160].

#### 3.3.3. Non-Coding RNA

Non-coding RNAs (ncRNAs) are functional RNAs that modulate gene expression post-transcriptionally, and their gene diversity is considered to be closely related to biological complexity. Such ncRNAs include microRNAs (miRNAs), circular RNAs (circRNAs), and long non-coding RNAs (lncRNAs). They are highly expressed in the brain and participate in the regulation of pathophysiological processes, including neural development and plasticity, cerebral ischemic injury, neurodegeneration, and other neurological diseases [161]. Increasing evidence suggests that ncRNAs can regulate inflammation in the brain by affecting microglial activation and polarization (Figure 1) through different mechanisms.

MiR-210, miR-155, and miR-342 have been shown to be relevant factors mediating pro-inflammatory pathways and M1-type activation [161,162]. Li et al. reported that miR-210-triggered the microglia M1 phenotype by targeting sirtuin-1 (SIRT1), thereby reducing deacetylation of NF-κB p65 in an animal model of neonatal rats [163]. MiR-155 has been shown to be a pro-inflammatory signal that activates TREM2-Apolipoprotein E (ApoE) signaling in microglia in inflammatory states [164,165]. MiR-342 can affect microglial activation and its crosstalk with neurons. Overexpression of miR-342 is sufficient to trigger the NF-kB pathway by inhibiting BCL2-associated athanogene-1 (BAG-1), resulting in enhanced IL-1β and TNF-α secretion in rat brain-derived mixed glial cells [166]. MiR-711 and miR-145 mediate neuroprotective signaling and M2 phenotype activation in primary murine microglia [167]. Exosomes play a crucial role in cell-to-cell communication by transporting bioactive miRNAs. Microglia secrete exosomes containing miRNAs that affect brain injury development. For example, cerebral ischemic injury in mice was alleviated by transporting exosomal miRNA-137 [168]. Upregulation of exosomal miRNA-124-3p expressed by BV2 microglia cells inhibits neuronal inflammation and promotes neurogenesis following traumatic brain injury [169].

CircRNAs are highly expressed in the CNS and are involved in various regulatory processes [170]. It has been reported that circular RNA can change the microglial polarization in central system diseases by regulating the activity of miRNA [171]. Circ-ubiquitin specific peptidase 10 (Usp10) may promote microglial activation by targeting miR-152-5p/CD84 and inhibiting the secretion of pro-inflammatory factors in BV2 microglia [172]. CircHivep2 interacts with miR-181a-5p to upregulate the expression of suppressor of cytokine signaling 2 (SOCS2), promote microglial activation, and inhibit the expression of pro-inflammatory factors in epilepsy [173]. CircPrkcsh regulates the MEKK1/JNK/p38 MAPK pathway through miR-488 to stimulate M1 polarization of microglia in vivo and in vitro [174]. Circ-Rps5 promotes M2 microglial polarization under conditions of hypoxia by affecting the downstream targets SIRT7 and miR-124-3p in MCAO mice [175].

LncRNAs can act as endogenous competing RNAs to regulate microglial polarization during central system injury. LncRNA-H19, as a classical lncRNA, could competitively bind to let-7b to promote an inflammatory response by targeting STAT3 and activating hippocampal glial cells in a rat model [176]. H19 knockdown blocked M1 microglial polarization driven by oxygen–glucose deprivation (OGD) and increased Arg-1 and CD206 production both in vivo and in vitro [177]. LncRNA-Gm4419, through phosphorylation of IκBα, results in transcriptional activation of IL-1β, IL-6, and TNF-α in the nucleus, which activates microglia during OGD/R injury [178]. LncRNA-maternally expressed gene 3 (Meg3) has been shown to play a crucial role in various biological processes. Meng et al. have shown that Meg3 attenuates microglial activation by targeting the miR-7a-5p/Nlrp3 pathway in vitro [179]. Overexpression of lncRNA-small nucleolar RNA host gene 14 (SNHG14) significantly promoted the activation of BV2 microglial cells induced by OGD and increased the production of TNF-α and nitric oxide (NO) [180].

## 4. Microglia Phagocytic Function following Stroke

### 4.1. Microglia Phagocytotic Function during Physiological State

Microglia are the main phagocytes in the brain, and they are capable of engulfing and digesting extracellular materials and other cells. Microglial phagocytosis is an important mechanism for the formation of neural circuits. During brain development, microglia form neural circuits by phagocytosing dendrites, axons, excess synapses, myelin, neurons, and neuronal precursors, and clearing proteins with high turnover, such as beta-amyloid (Aβ) [181,182].

In healthy brain, microglia are in a resting state with highly differentiated morphologies and elongated processes that are capable of recognition and phagocytosis [8]. Time-delay imaging has shown that microglial processes are highly active even in uninjured brain and their contacts with synapses are transient but frequent [183]. Microglia play a crucial role in monitoring synapses in the physiological state and participate in the maturation or elimination of synapses [79]. In the early stages of brain development, too many synapses are formed, and the number of synapses decreases over time; in adulthood, the synaptic density remains constant. Microglia phagocytosis plays an important role in this process, engulfing synapses with weak neural activity, which is called developmental synaptic reduction [184,185]. A large number of neuronal connections can be removed by synaptic pruning and loss or shortening of axons and dendrites. This leads to a broader reorganization of neuronal structures and their connections, which is closely related to the formation of neural circuits and various functional gains in humans [79,181].

In addition to selectively removing synapses from viable neurons, microglia can phagocytose viable neuronal precursors and structures during development. In monkey and rodent models, microglia phagocytize neural precursors in multiple regions of the developing CNS. The number of neural precursors can be significantly increased by deactivating microglia with tetracycline in rat uterus or by removing them from the fetal cerebral cortex with liposomal clodronate, whereas the number can be reduced through maternal immune activation of microglia in the uterus [186]. Microglia can also phagocytose glial precursors during cortical development. It follows that microglia can limit excessive cell production and ultimately regulate the cerebral cortex and other CNS structures [186].

### 4.2. Functional Changes of Microglia Phagocytosis after Stroke

**Figure 3:**
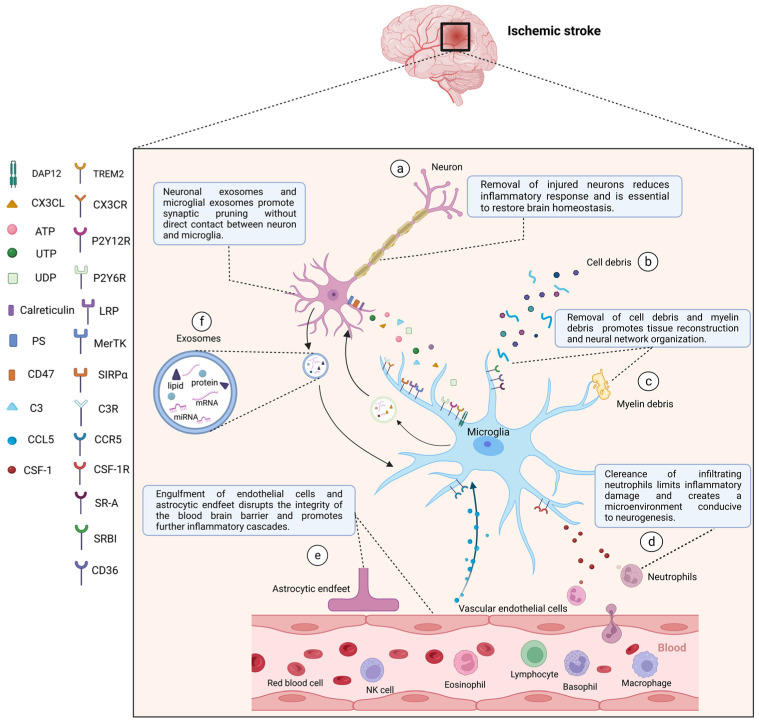
After stroke, activated microglia mediate phagocytosis in the central nervous system through a combination of ligands expressed by target cells and corresponding receptors in microglia. After stroke, activated microglia can directly phagocytose damaged or dead neurons (**a**), cell debris (**b**), myelin debris (**c**), and peripheral neutrophils (**d**) to reduce inflammatory responses, create a microenvironment for tissue repair, and restore brain homeostasis. Microglia can also phagocytose vascular endothelial cells and astrocytic endfeet (**e**) to damage the integrity of the blood-brain barrier and exacerbate the inflammatory development. In addition, neuronal exosomes and microglial exosomes can promote synaptic pruning (**f**). (**a**) Injured neurons can express “Find me” signals, including CX3CL, ATP, UTP, and UDP, recognized by corresponding microglial receptors including CX3CR, P2Y12R, P2Y6R, and “eat me” signals including PS, calreticulin, C3 recognized by LRP, MerTK, and C3R respectively. “Do not eat me” signal (CD47) can be downregulated, and its receptor is SIRPα. (**d**) Neutrophils release CSF-1, which is recognized by CSF-1R in microglia. (**e**) Vascular endothelial cells secrete CCL5, which binds to CCR5 in microglia to play a protective role in maintaining the integrity of the blood-brain barrier. Other microglial surface receptors, including TREM2, SR-A, SR-BI, CD36, are involved in the clearance of apoptotic cells as well as cell and myelin debris. CX3CL, C-X3-C motif chemokine ligand; CX3CR, C-X3-C motif chemokine receptor; ATP, adenosine triphosphate; UTP, uridine triphosphate; UDP, uridine diphosphate; P2Y12R, P2Y12 receptor; P2Y6R, P2Y6 receptor; PS, phosphatidylserine; MerTK, c-mer tyrosine kinase; C3, complement 3; C3R, C3 receptor; LRP, low-density lipoprotein receptor-associated protein; SIRPα, signal regulatory protein α; CSF-1, colony-stimulating factor-1; CSF-1R, CSF-1 receptor; CCL5, C-C motif chemokine ligand 5; CCR5, C-C motif chemokine receptor 5; TREM2, triggering receptor expressed on myeloid cells 2; DAP12, DNAX activation protein 12; SR-A, scavenger receptor A; SR-BI, scavenger receptor class B type I. Created with BioRender.com.

In immune inflammation and stroke recovery, microglial phagocytosis is a double-edged sword. Microglia phagocytose stressed-but-viable and damaged neurons, leading to excessive cell death and neurological deficits, and they phagocytose endothelial cells and astrocytic endfeet, leading to destruction and leakage of the BBB. However, the removal of dead neurons and tissue debris, such as cell body debris and myelin debris, promotes tissue reconstruction and neural network reorganization to a certain extent [187,188,189,190]. The removal of infiltrating neutrophils can create a microenvironment that is conducive to neurogenesis, limits inflammatory damage, and promotes tissue repair (Figure 3).

Stroke is often accompanied by cell death and infiltration of blood-derived monocytes into the brain parenchyma. The accumulation of dead cells and cell debris causes an excessive inflammatory response; therefore, removal is essential for restoration of brain homeostasis, and both microglia and macrophages are involved in this process. However, studies have shown that microglia participate more in the phagocytosis of dead cells within the CNS [191]. After MCAO, microglia, as phagocytes, accumulated prior to blood-derived monocytes in the infarct zone. On the first day, the morphology of the microglia changed to an ameboid or round shape and acquired the ability to phagocytose. Subsequently, their numbers continue to increase to become the main phagocytes in the early stages of stroke. On day 7, the density of microglial infiltration was approximately three times higher than that of macrophages. By the time monocytes infiltrate the brain tissue, the dead cells are almost completely removed. Studies have shown that infiltration of blood-derived macrophages into the infarct area is delayed by at least 24–48 h after stroke onset [91,192].

In the ischemic core area, there are more dead neurons owing to more severe ischemia and hypoxia. Decomposition of dead cells further leads to the release of toxic substances and self-antigens and enhances the autoimmune response. Microglia begin to infiltrate and phagocytose within a few hours. This process contributes to the resolution of inflammation. In the penumbra area, there are more stressed neurons, which are damaged or have changes in activity after stimulation but are still salvageable. Phagocytosis of these neurons is unfavorable and may lead to exacerbation of brain atrophy and dysfunction [193].

M2-like microglia have a persistent capacity to remove debris after ischemic stroke. The studies to date have mostly focused on multiple sclerosis for the clearance of myelin debris. Phagocytosis of damaged myelin by microglia improves the immune microenvironment for oligodendrocyte differentiation and plays an important role in remyelination and white matter recovery.

Neutrophils from the blood system rapidly infiltrate the brain parenchyma after ischemia, releasing pro-inflammatory factors, metalloproteinase matrix, and oxygen free radicals to mediate inflammatory damage and enhance neurotoxic effects. Microglia can efficiently phagocytose these cells. In time-lapse images of hippocampal culture sections from an OGD model, microglia migrated to and phagocytosed apoptotic and surviving neutrophils [194], which was also observed using two-photon microscopy in a rat model of cerebral ischemia [195].

The BBB is crucial for maintaining brain homeostasis. After stroke, BBB disruption can occur throughout the brain owing to the infiltration of immune cells, thereby promoting further inflammatory cascades [196]. Microglia can interact with endotheliocytes and regulate the BBB [197]. On the one hand, microglia can directly engulf astrocytic endfeet and disrupt the integrity of the BBB. On the other hand, microglia aggravate endothelial necrosis and exacerbate BBB disruption by secreting pro-inflammatory cytokines such as IL-1β, IL-6, and TNF-α. IL-1β released from microglia downregulates the production of claudin-5, occludin, and zonula occludens (ZO)-1, thereby increasing BBB permeability [198].

In addition, microglia can act indirectly as synaptic regulators without physical contact with neurons [199]. Microglia exposed to inflammation secrete extracellular vesicles (EVs) that contain proteins, lipids, and RNAs. Internal miR-146a-5p targets and inhibits the expression of presynaptic synaptotagmin1 (Syt1) and postsynaptic neurolectin 1 (Nlg1), resulting in a reduction in the number of dendritic spines and the density of excitatory synapses [200]. Neuronal exosomes promote microglial phagocytosis and enhance synaptic pruning. Incubation of microglia with neuron-secreted exosomes upregulated the expression of complement 3 (C3) in microglia and enhanced microglial clearance of inappropriate synapses [201].

In stroke-mediated inflammation, the mechanism of microglial phagocytosis is affected by many factors, including age, sex, physiological condition, lesion site, phagocytic target, various types of phagocytic cells (astrocytes, pericytes, monocytes, peripherally infiltrated neutrophils, etc.), and the timing and ability of phagocytosis [191,202]. Therefore, the comprehensive mechanism and effect of microglia in case conditions need to be studied further.

### 4.3. Phagocytic Signaling

Whether a target cell is engulfed depends on the signals expressed and released. Under normal physiological conditions, microglia mainly phagocytose naturally apoptotic cells, and their phagocytic function maintains a steady balance. Under inflammatory conditions, when neurons are damaged or dead, their surface will be exposed to “find me” and “eat me” signals, and the expression of “do not eat me” signals will be downregulated, inducing chemotaxis and phagocytosis of microglia [189,203]. Several recognized surface signals expressed by neurons and other cells that mediate phagocytosis are described below (Figure 3).

#### 4.3.1. Find Me Signal

In the CNS, C-X3-C motif chemokine ligand (CX3CL) is expressed and released by neurons, and specifically binds to the receptor CX3CR1 on the surface of microglia [133]. The CX3CL1/CX3CR1 biological axis is involved in the directed migration of chemotactic cells as well as immune and inflammatory responses. Studies have shown that CX3CL1 inhibits LPS-induced microglial activation by limiting the release of pro-inflammatory factors in vitro and in vivo, which results in a neuroprotective effect [204]. Additionally, CX3CR1 activation is important for microglial migration to lesion sites during development. It has been demonstrated that mice lacking CX3CR1 exhibit a variety of neuronal defects, which is thought to be associated with defective phagocytosis of microglia [205]. CX3CL1/CX3CR1 contribute significantly to synaptic pruning by microglia. CX3CR1 knockout mice also exhibited decreased microglial numbers during postnatal development.

Nucleotides, including adenosine triphosphate (ATP) and uridine triphosphate (UTP), can be released as transmitters by active neuron synapses or by pannexin-1 channels of apoptotic, damaged neurons [203]. Microglial recruitment to neuron is primarily mediated through activation of P2Y12 receptor (P2Y12R). During CNS development, P2Y12R knockdown delays synaptic pruning by microglia in the mouse visual cortex [206]. Release of ATP/ADP is an important condition for inducing the directional and rapid migration of microglia to injured neurons. In an animal study, the application of apyrase, which is capable of degrading extracellular ATP/ADP, inhibited microglial motility and migration to injured neurons. ATP/ADP-dependent microglial migration facilitates the clearance of dying and dead cells and alleviates secondary damage within a critical time after nervous damage [207,208].

In addition, in vivo and in vitro studies have shown that the metabotropic P2Y6 receptor (P2Y6R) expressed in microglia can also trigger phagocytosis when activated by uridine diphosphate (UDP) [209]. Pharmacological inhibition of P2Y6R can effectively prevent microglial phagocytosis by neurons [210,211].

#### 4.3.2. Eat Me Signal

Phosphatidylserine (PS) can be irreversibly exposed on the surface of apoptotic cells, becoming a critical “eat me” signal [212]. Phosphatidylserine is normally restricted to the cell membrane [213]. Upon apoptosis, PS eversion occurs due to the decline in phosphoserine aminotransferase activity, which is a general feature of apoptotic cells and an important signal for recognition by phagocytes [214]. The cause of eversion is related to oxidative stress, increased calcium levels, and glutamate release following neuronal injury. During inflammation, phosphatidylserine binds to the microglia-released opsonin milk fat globule-epidermal growth factor 8 (MFG-E8). It is then recognized by the vitronectin receptor (VR) on the microglial surface [203,215,216,217] and plays an important role in inducing phagocytosis. The combination of opsonin, such as growth arrest-specific protein 6 (GAS6), galectin-3 (Gal-3), and Tubby with “eat me” signal on the surface of neurons can activate the microglia surface receptor c-mer tyrosine kinase (MerTK) [218,219]. The upregulation of MFG-E8 and MerTK has been reported to be delayed by 2 to 3 days after focal cerebral ischemia in an animal model [220], which may be consistent with the resolution of inflammation. Thus, the specific binding of phosphatidylserines to opsonins and the expression of their receptors results in the detection and phagocytosis of signal-exposed neurons, a process that contributes to inflammatory response [193].

Calreticulin, normally located in the endoplasmic reticulum (ER), is released to the neuronal cell surface in response to stress or inflammatory signals. Calreticulin acts as an “eat me” signal or opsonin to trigger phagocytosis through low-density lipoprotein receptor-associated protein (LRP) on the surface of microglia [181,188]. The addition of nanomolar calreticulin could result in attraction of microglia, stimulation of microglia to release pro-inflammatory factors, alteration of microglial morphology and proliferation, and promotion of phagocytosis [188,221]. LPS-induced microglial phagocytosis can be inhibited by calreticulin or microglial surface LRP1 receptor blocker [222].

#### 4.3.3. Do Not Eat Me Signal

CD47 is a transmembrane protein that is expressed on neurons and inhibits phagocytosis by binding to signal regulatory protein α (SIRPα) on phagocytes. It can serve as a “Do not eat me” signal [181,223]. CD47 or SIRPα knockdown resulted in increased microglial synaptic phagocytosis of neurons, indicating that the CD47-SIRPα signaling pathway plays an important role in regulating synaptic elimination [224]. In addition, according to an animal study, CD47 expressed on myelin phospholipid debris can inhibit microglial phagocytosis by binding to SIRPα. Eliminating SIRPα-dependent inhibition of phagocytosis can promote the removal of myelin debris and advance functional recovery from nerve injury [225].

There is a high density of sialic acid residues on glycoproteins and glycolipids on the surface of the neuronal membranes. The sialylated protein can activate sialic acid-binding immunoglobin-like lectins (Siglecs) including Siglec-11(in humans) and Siglec-E (in mice). In addition, it can also inhibit the phagocytosis of microglia by inhibiting the binding of the opsonins C1q, C3, and Gal-3 [226,227]. Reduced sialylation has been shown to disrupt synaptic homeostasis and damage neurons in middle-aged mice [228]. In addition, sialidase treatment of BV-2 microglia can induce IL-6 release and activate TLR4 signaling. These results show that sialic acid plays an important role in regulating microglial activation and mediating the inflammatory response in the nervous system [229].

Excluding the phagocytosis of neurons, microglia can migrate, engulf and digest other extracellular materials, including endothelial cells, immune cells, and myelin debris [91]. Studies have shown that systemic inflammation can induce the release of C-C motif chemokine ligand 5 (CCL5) by endothelial cells, which attracts microglia in the CNS to migrate to the cerebrovascular system. In vivo and in vitro experiments have shown that the CCL5/C-C motif chemokine receptor 5 (CCR5) signaling pathway helps attract microglia to blood vessels and induces microglia to express the tightly connected transmembrane protein claudin-5 (CLDN5). Subsequently, microglia permeate through the neurovascular unit, contacting endothelial cells, and forming tight connections to maintain the integrity of the BBB [230,231]. Ablation or blocking of the CCL5 signaling pathway increases BBB permeability.

Phagocytosis of peripheral neutrophils by reactive microglia after ischemic stroke is thought to be closely related to the surface receptor colony-stimulating factor-1 receptor (CSF-1R). CSF-1R is a tyrosinase that binds to signal molecules such as PI3K and AKT and induces ERK1/2-mediated signal transduction in microglia [232,233,234]. In a mouse model of MCAO, long-term treatment with CSF-1R antagonists led to a decrease in the number of microglia, an increase in the number of neutrophils, and an expansion of the ischemic lesion, which adversely affected local tissue recovery [115].

The scavenger receptor (SR) family, including food-borne receptor class A (SR-A, CD204), scavenger receptor class B type I (SRBI), and CD36 are cell surface proteins involved in the clearance of cell debris, myelin, bacteria, apoptotic cells, and outer rod segments [235]. SR-A expressed on neonatal microglia regulates the phagocytosis of apoptotic cells expressing phosphatidylserine [236]. In AD, activated microglia phagocytose Aβ via scavenger receptors [237]. CD36 is essential for the removal of myelin debris in neuroinflammation [238].

## 5. Therapeutic Intervention Targeting Microglia in Stroke Rehabilitation

Microglia can regulate neuroplasticity after stroke and play a critical role in the recovery from neuroinflammation. Endogenous microglial activation or polarization may not be sufficient to achieve the desired effects of structural and functional recovery. Therefore, exogenous therapy targeting microglia can be used to promote post-stroke recovery.

### 5.1. Pharmacotherapy

The use of drugs can provide a beneficial microenvironment for microglial activation and polarization, thus promoting the positive regulation of microglia during inflammation (Table 3). The most commonly used drug is minocycline. Due to its high lipophilicity, minocycline can cross the BBB and inhibit microglial activation. The mechanism is as follows: First, it can prevent microglial activation by attenuating the NLRP3 inflammasome signaling pathway to ameliorate ischemic brain damage [239]. Second, the expression of pro-inflammatory factors such as IL-1β and TNF-α is decreased, and the expression of anti-inflammatory factors such as IL-10 and TGF-β is increased in microglia around the infarction area [240]. Third, minocycline can affect the STAT1/STAT6 pathway to inhibit M1 polarization of microglia and promote M2 polarization. In addition, early minocycline treatment reduces microglial phagocytosis [241].

Many drugs can affect M2-like polarization by regulating microglial inflammatory cytokines. Wnt-3a [242] reduces the expression of iNOS, TNF-α, and CD16/32, increases the expression of CD206 and Arg-1, and changes the microglial polarization state from M1 to M2. Delayed treatment with recombinant Gal-3 after stroke is related to the proliferation of chitinase-like protein 3-positive microglia and decreased iNOS expression [243]. Atorvastatin can reduce the expression of IL-6, TNF-α, and monocyte chemotactic protein (MCP)-1, and increase the production of IL-10 after stroke. These results suggest that it can effectively alleviate microglia-mediated neuroinflammation after stroke [244]. Exendin-4, an agonist of the glucagon-like protein-1 (GLP-1) receptor, had no effect on M1 markers in microglia but increased the expression of M2 markers [245]. Bendavia is a mitochondria-targeted tetrapeptide that reduces the expression of MMP-9 and TNF-α [246].

In addition, many drugs regulate microglia by directly or indirectly affecting transcription factors: It has been shown vx-765 [247], baicalein [248] and cottonseed oil [249] alter the phenotype by switching polarization to M2-like microglia. This is associated with inhibition of NF-κB activation [250]. Gardenia extract GJ-4 inhibits microglia-mediated neuroinflammatory responses by inhibiting JAK2 and STAT1 pathways [251]. Studies have shown that melatonin can activate the STAT3 pathway to promote microglial polarization toward the anti-inflammatory phenotype, thus inhibiting the neurotoxic effect of pro-inflammatory microglia on OGD neurons [252]. Ki20227, a specific inhibitor of CSF-1R, can downregulate the NLRP3 pathway and inflammasome activation, thereby reducing the microglial number and significantly reducing dendritic spinous loss and behavioral deficits following transient global cerebral ischemia [253,254]. Curcumin not only reduces the expression of pro-inflammatory factors and promote M2 microglial polarization after stroke [255] but also suppresses microglial pyroptosis and ameliorates stroke-induced white matter injury by inhibiting the NF-κB/NLRP3 signaling pathway [256]. Traditional Chinese medicine extracts such as salidroside [257], tripterine [258], resveratrol [259] and schisandrin B [260] have been shown to regulate microglial anti-inflammatory polarization and play a protective role after stroke in animal models.

**Table 3:** Pharmacotherapy can promote post-stroke recovery by targeting microglia. ↑, upregulate; ↓, downregulate; Gal-3, galectin-3, MCAO/R, middle cerebral artery occlusion/reperfusion; tMCAO, transient MCAO; dMCAO, distal MCAO; pMCAO, permanent MCAO; PT, photothrombosis; OGD/R, oxygen-glucose deprivation/reperfusion; LPS, lipopolysaccharide; IL-1β, interleukin-1β; IL-18, interleukin-18; NLRP3, NOD-like receptor thermal protein domain associated protein 3; TNF-α, tumor necrosis factor-α; IL-10, interleukin-10; TGF-β, transforming growth factor-β; Ym1, chitinase-like protein 3, IL-6, interleukin-6; iNOS, inducible nitric oxide synthase; Arg-1, arginase-1; IFN-γ, interferon-γ; IL-17, interleukin-17; IL-4, interleukin-4; MCP-1, monocyte chemotactic protein-1; MMP9, matrix metalloproteinase 9; NO, nitric oxide; COX-2, cyclooxygenase-2; STAT1, signal transducer and activator of transcription 1; STAT6, signal transducer and activator of transcription 6; NF-κB, nuclear factor kappa-B; TLR4, toll-like receptor 4; STAT3, signal transducer and activator of transcription 3; JAK2, Janus kinase 2.

Drug	In Vivo		In Vitro		Effect on Microglia	Signal	Reference
	Animal Model	Treatment	Cell Culture	Treatment			
Minocycline	Mouse (tMCAO)	Intraperitoneal injections after MCAO induction; 10, 25, and 50 mg/kg/day for 3 consecutive days	BV2 microglial cells oxygen–glucose deprivation/reoxygenation (OGD/R) cell model	Minocycline at doses of 0.01, 0.1, 1, 10, and 100 μM; preincubated 1 h before OGD/R injury	IL-1β↓, IL-18↓, NLRP3↓		[239]
	Rats (tMCAO)	Intravenous injection after reperfusion onset; a single dose (3 mg/kg)			IL-1β↓, TNF-α↓, IL-10↑, TGF-β↑, Ym1↑		[240]
	Mouse (MCAO/R)	Intraperitoneal injections after reperfusion; 10, 25, or 50 mg/kg/day for 2 weeks	Primary microglia from cerebral cortex of newborn mice	LPS (100 ng/mL) + IFN-γ (20 ng/mL) + minocycline (50 μM); incubation for 24 h	IL-1β↓, IL-6↓, iNOS↓, TNF-α↓, Arg-1↑, IL-10↑, TGF-β↑, Ym1↑	STAT1/STAT6 pathways ↓	[241]
Wnt-3a	Mouse (tMCAO)	Intranasally delivered at the time of reperfusion and next 2 days; 2 μg/kg/day			iNOS↓, TNF-α↓, Arg-1↑, CD206 ↑		[242]
Gal-3	Mouse (MCAO)	Intracortical injection at 24 h following MCAO; 100 ng/mouse (20–25 g)	Primary cell cultures from the brains of the adult, 8–9-week-old C57BL/6 wild-type mice	Incubation with Gal-3 (5 μM) for 24 h	TNF-α↓, IL-1β↓, IFN-γ↓, IL-17↓, iNOS↓, Ym1↑, IL-4 ↑, IL-6 ↑		[243]
Atorvastatin	Mouse (pMCAO)	Oral gavage after MCAO induction; 20 mg/kg/day			IL-6↓, TNF-α↓, MCP-1↓, IL-10 ↑		[244]
Exendin-4	Mouse (MCAO)	Intraperitoneal injection; 50 mg/kg at 1.5 h after MCAO induction; 0.2 mg/kg daily for 3 days until sacrifice	Primary microglia-enriched cultures were prepared from whole brains of 2- to 3- day-old mice	LPS (10 ng/mL) + Ex-4 (40 ng/mL); incubation for 24 h	CD206↑, Arg-1↑, Ym1/2↑		[245]
Bendavia	Mouse (tMCAO)	Intraperitoneal injection immediately after reperfusion and 4 h later; 5 mg/kg			MMP9↓, TNF-α ↓		[246]
Vx-765	Mouse (MCAO)	intraperitoneal injection starting immediately after MCAO induction; 50 mg/kg for 3 consecutive days			IL-1β↓, TNF-α↓, iNOS↓, TGF-β↑, Ym1↑	NF-κB signaling↓	[247]
Baicalein	Mouse (MCAO)	Intragastrical administration after reperfusion; 100 mg/kg/day for 3 days	BV2 microglial cells	LPS (100 ng/mL) + IFN-γ (20 ng/mL) + baicalein (45 μM); incubation for 24 h OGD stimulated BV2 cells + baicalein (45 μM); incubation for 24 h	Ym1/2↑, Arg-1↑, CD206↑, TNF-α↓, IL-1β↓, IL-6↓, NO↓	TLR4/NF-κB ↓, phosphorylated STAT1 ↓	[248]
Cottonseed oil	Rats (MCAO/R)	Subcutaneous injection before MCAO; 1.3 mL/kg/day for 3 weeks			IL-1β↓, IL-6↓, TNF-α↓	TLR4/NF-κB ↓	[249]
GJ-4	Rats (MCAO/R)	Oral administration after MCAO induction; 10, 25, 50 mg/kg/day for 12 days			iNOS↓, COX-2↓, MMP9↓	JAK2/STAT1 ↓	[251]
Melatonin	Mouse (dMCAO)	20 mg/kg at 0 and 24 h after reperfusion	Co-culture of BV2 cells (growing on culture inserts) and OGD neuron	Melatonin (100, 200, or 400 mM); incubation for 12 h	pro-inflammatory markers ↓, anti-inflammatory markers ↑	p-STAT3/STAT3↑	[252]
Ki20227	Mouse (PT)	Oral gavage before modeling; 0.002 mg/kg/day for 7 consecutive days			TNF-α↓, iNOS ↓, IL-10↑, Arg-1 ↑, NLRP3↓, Active caspase 1 ↓	NF-κB signaling↓	[253]
Curcumin	Mouse (tMCAO)	Intraperitoneal injection; 150 mg/kg at 0 h and 24 h after reperfusion	BV2 microglial cells	LPS (100 ng/mL) + IFN-γ (20 ng/mL) + curcumin (12.5 and 25 µmol/L); incubation for 48 h	TNF-α↓, IL-12p70↓, IL-6 ↓		[255]
	Mouse (MCAO/R)	Intraperitoneal injection; 150 mg/kg/day for 7 days after ischemic stroke	Primary microglia were isolated from the whole brains of neonatal C57BL/6J mice	LPS (100 ng/mL) + curcumin (12.5 μM); incubation for 24 h		NLRP3/NF-κB pathway ↓	[256]

### 5.2. Exercise

Exercise can promote the recovery of inflammation after stroke by affecting the activation and function of microglia, and the mechanism mainly depends on the regulation of pro-inflammatory and anti-inflammatory cytokines (Table 4). Exercise can promote the production of the anti-inflammatory cytokines IL-4 and IL-10, which can interact with microglial receptors and inhibit CNS inflammation [261,262,263,264]. In addition, exercise can downregulate the expression of pro-inflammatory factors and inhibit microglial activation in human and mouse models. Long-term voluntary exercise can reduce the levels of IL-1β, iNOS, and IL-6 [265,266], downregulate the TLR pathway, and reduce the activation of hippocampal microglia [267].

Studies have found that different intensities of treadmill exercise training can inhibit the expression of NLRP3 inflammasome components, stimulate the expression of endogenous BDNF, reduce the levels of pro-inflammatory factors, and upregulate the levels of anti-inflammatory factors [268]. Colocalization analysis showed that M2-type microglia increased and M1-type microglia decreased in the infarct area, and their morphology also changed significantly, from ameboid to the branched form. High-intensity interval training (HIIT) improves functional recovery after ischemic stroke better than moderate-intensity continuous training (MICT) [269]. Thus, the anti-inflammatory effect of exercise can inhibit inflammation-mediated pyroptosis by polarizing microglia towards a neuroprotective M2 phenotype [270]. In addition, it can also promote the recovery of brain plasticity and function by upregulating the level of neuro-nutrients and enhancing synaptic generation [268,269]. In terms of exercise time, studies have found that more than four consecutive weeks of treadmill training can effectively reduce the activation of microglia compared to one week of training, but it had no effect on neuroprotection [265,271].

Exercise may also regulate synaptic plasticity by promoting the migration of exosomes into the brain [272] through circulation and by preventing the overactivation of microglia [273]. Exosomes have been shown to inhibit the overactivation of M1-type microglia [274,275], increase the complexity of dendrites and expression of synaptic plasticity-associated proteins, and significantly reduce the volume of cerebral infarction and dysfunction.

In addition to aerobic exercise, such as running, training of damaged forelimbs in post-stroke mice significantly reduces excessive microglial activation in the area around the lesion [276,277].

For hemorrhagic stroke, 8 days after intracerebral hemorrhage induction, exercise preconditioning mice showed reduced lesion volume and increased pro-survival factors in plasma, and promoted the recovery of neurological deficits and induced microglial phagocytic function [278].

However, the studies to date on the regulatory effects of exercise on microglial activation have mostly focused on PD and AD [279], and further studies are needed on the post-stroke-related mechanisms of inflammation regulation.

**Table 4:** Exercise and NIBS as the main rehabilitative interventions can promote post-stroke recovery by targeting microglia. ↑, upregulate; ↓, downregulate; MCAO, middle cerebral artery occlusion; dMCAO, distal MCAO; PT, photothrombosis; rTMS, repetitive transcranial magnetic stimulation; tDCS, transcranial direct current stimulation; TUS, transcranial ultrasound stimulation; tFUS, transcranial focused ultrasound stimulation; TBS, theta-burst stimulation; HIT, high-intensity; MOD, moderate-intensity; IL-4, interleukin-4; NLRP3, NOD-like receptor thermal protein domain associated protein 3; IL-10, interleukin-10; p75NTR, P75 neurotrophin receptor; BDNF, brain-derived neurotrophic factor; IFN-γ, interferon-γ; Gal-3, galectin-3; Syn, synaptophysin; PSD-95, postsynaptic density protein 95; TGF-β, transforming growth factor-β; VEGF, vascular endothelial growth factor; HIF-1α, hypoxia-inducible factor-1α; TLR4, toll-like receptor 4; NF-κB, nuclear factor kappa-B; STAT6, signal transducer and activator of transcription 6; GABA, γ-aminobutyric acid; IL-10R, interleukin-10 receptor.

	Type	Treatment (Intensity, Time, Frequency, Duration)	Model	Effect on Microglia	Outcome	Reference
Exercise	Treadmill exercise	12 m/min; 30 min/day; 3 or 6 consecutive days	Rats (MCAO)	IL-4↑ M1-like markers↓ M2-like markers↑	Improving neurobehavioral outcomes	[270]
		5–6 m/min; 5 min/day; 3 consecutive days	Mouse (MCAO)	NLRP3↓	Showing better improvements at functional levels	[268]
		HIT program: 10 days (>25 m/min) MOD program: 2 days (<20 m/min)	Rats (MCAO)	IL-10↑, p75NTR↑, BDNF↑	Promoting cerebral plasticity	[269]
		30 min/day; 5 days/week; 4 weeks	Mouse (MCAO)	Iba1+↑ (hippocampal CA1 region)	Alleviating increased neuroinflammation	[271]
		25 cm/s; 30 min/day; 3 days/week 4.5 weeks	Mouse (MCAO)	IL-10↑, NLRP3↑, IFN-γ↑, Gal-3↓ (caused by stress)	Having beneficial neuro-inflammatory effects; inducing detrimental stress response by forced running	[280]
		10 m/min; 60 min/day; 5 weeks	Mouse (microinjection of collagenase into the striatum region)	CD36/Iba1-double positive cells↑	Contributing to neuroprotection	[278]
		12 m/min; 30 min/day; 5 times/week; 4 weeks	Rats (MCAO) (exosomes injection)	Excessive microglial activation↓, Syn↑, PSD-95↑	Regulating synaptic plasticity and protecting neural function	[273]
	Skilled reaching training of the impaired forelimb	5 days/week; 14 or 42 days	Rats (PT)	Excessive microglial activation↓	Modulating perilesional cellular plasticity and contributing to a better functional recovery	[276]
		10 or 42 days	Rats (PT)	Excessive microglial activation↓	Improving functional recovery	[277]
rTMS	Continuous TBS	5 min (3 pulses of 50 Hz repeated every 200 ms); 5 days	Rats (PT)	Pro-inflammatory cytokines↓	Improving the local neuronal microenvironment	[281]
		5 min (3 pulses of 50 Hz, repeated every 200 ms); 6 days	Rats (PT)	TGF-β↑, VEGF↑, HIF-1α↑	Presenting protective effects in the context of ischemic stroke; contributing to vascular repair and protection	[282]
	Intermittent TBS	Ten 50 Hz bursts with 3 pulses each repeated 20 times at 5 Hz intervals; twice per day; 7 continuous days	Mouse (MCAO)	TLR4/NF-κB/NLRP3 signaling pathway↓	Alleviating locomotor deficits and neuronal pyroptosis	[283]
	High frequency	10 Hz rTMS with a total of 60 trains; 20 pulses per train (1200 pulses); 10 s intertrain interval; for 11 min 44 s	Rats (MCAO)	NF-κB↓, STAT6↓	Promoting neurogenesis and improving neural function recovery	[284]
tDCS	Cathodal	500 µA, 15 min; once per day; 10 days	Rats (MCAO)	Iba1+↓ Pro-inflammatory factors↓ Anti-inflammatory factor↑	Accelerating recovery from neurologic deficit and brain damage	[285]
		250 µA; 40 min; 1 day	Mouse (PT)	CD206↑ CD68↓	Being effective from a functional point of view	[286]
		250 µA; 40 min	Mouse (MCAO)	Iba1+↓ GABA and glutamate↓	Exerting a measurable neuroprotective effect	[287]
	Anodal	250 µA; 15 min; 10 days	Mouse (MCAO)	Iba1+↓	Inducing regeneration and promoting functional recovery	[288]
	Cathodal or anodal	250 µA (110.13 A/m^2^) or 500 µA (220.3 A/m^2^); 15 days	Mouse (PT)	CD16/32↓, Iba1+↓	Impacting neurogenesis and influencing functional recovery	[289]
TUS/ tFUS	Low intensity	528 mW/cm^2^; 5 days; 15 min/day; 5 days before MCAO	Mouse (MCAO)	VEGF↑, BDNF↑, Caspase-3↓	Ameliorating brain damage	[290]
		86 mW/cm^2^; 60 min	Rats (dMCAO)	Inflammatory factors↓	Increasing cerebral blood flow and supporting neuroprotection	[291]
		0.5 MHz; 120 mW/cm^2^; 7 consecutive days;	Mouse (MCAO)	M2 microglia↑ IL-10 and IL-10R↑	Promoting neurorehabilitation	[292]

### 5.3. Cell-Based Therapy

Cell-based therapy is considered a new potential therapeutic strategy, and its mechanisms include protective factor release, immune regulation, cell differentiation, and neural circuit reconstruction.

Stem cells have anti-inflammatory and immunomodulatory properties [293]. In vitro and in vivo studies have shown that bone marrow mesenchymal stem cells (BMMSCs) can migrate to ischemic sites and differentiate into neurons, endothelial cells, and glial cells. They can also produce and release trophic factors (such as BDNF, VEGF, GDNF, and TGF) that have neuroprotective effects [294]. The injection of human mesenchymal stromal cells (hMSCs) into adult mice has been shown to induce the activation of M2-type microglia [295], which play an important role in functional recovery, angiogenesis, and endogenous neurogenesis [296,297]. Adipose-derived stem cells have similar functions [298]. After intravenous injection of human umbilical tissue-derived cells (hUTC) into monkeys, the total density of activated microglia increased significantly in both the perifocal gray matter and subfocal white matter, which played a protective role in the effective clearance of debris in the peri-infarct area and enhancement of neuroplasticity [299].

Transplanted NSCs can generate both neurons and glial cells. Exogenous NSCs can be derived from induced pluripotent stem cells (iPSCs), embryonic stem cells , fetal tissue, and the adult nervous system and can also stimulate SVZ neurogenesis. NSC transplantation can reduce microglia, infiltrated macrophages, and cells expressing iNOS and cyclooxygenase-2 (COX-2) in rodent models [300]. Animal studies on NSC transplantation can be used as a multifaceted neurosupportive strategy in the acute/subacute phase of ischemic stroke to limit the severity of cell damage caused by ischemic injury [301].

Transplantation of iPSCs-derived microglia is an important component of translational neuroimmunology and has great therapeutic potential. Stem cells secrete a variety of bioactive molecules through paracrine signaling, which is involved in inflammation development, angiogenesis, and regeneration [302]. Human induced pluripotent stem cell-derived glial progenitor cell (GPCs)-conditioned medium produces high levels of neurotrophic factors [303]. An experiment performed in mice showed that iPSC-derived microglia can be transplanted into the brain via the transnasal route, thus providing a potential treatment approach [304].

The transplantation of exogenous microglia may also have a protective effect against ischemic brain injury. Exogenous microglial cells injected into the subclavian artery of gerbils can promote neuronal survival by migrating to CA1 vertebral neurons, increasing BDNF expression in the ischemic hippocampus, and preventing ischemia-induced neuronal deformation [305,306].

T_reg_ cells, a small subgroup of CD4+ T cells defined by the expression of marker proteins such as forkhead box protein 3 (Foxp3) and CD25 [307], have been found to be targets for neural repair in stroke recovery. According to in vitro and in vivo experiments, brain-infiltrated T_reg_ cells have a strong immunomodulatory effect on microglia to enhance their repair activity [308]. T_reg_ cells begin to infiltrate the infarcted area of mouse brain early after ischemic injury (days 1–7). As the disease progresses, the number of T_reg_ cells in the brain increases and remains high for at least one month after stroke [309]. Co-culture studies of T_reg_ cells and microglia showed that microglia genes associated with the anti-inflammatory phenotype were upregulated, such as arginase 1 (*Arg1*), fibrinogen like 2 (*Fgl2*), mannose receptor C-type 1 (*Mrc1*), interleukin 1 receptor antagonist (*Il1rn*), and galectin 3 (*Lgals3*). It was speculated that the interaction between T_reg_ cells and microglia enhances the neuroprotective effect of microglia and promotes the recovery of white matter in the chronic stage of stroke [308]. Animal studies have shown that ischemic brain injury can be attenuated effectively by promoting the increase in endogenous T_reg_ cells, such as by using CD28SA, poly(ADP-ribose) polymerase 1 (PARP1) inhibitors, IL-2/IL-2 antibody complex, etc. [310,311]. Treatment with exogenous T_regs_ at 2, 6, or 24 h after ischemia can reduce the volume of cerebral infarction and neurological deficits in mice [312]. Both in vivo and in vitro studies have demonstrated that exogenous T_regs_ can effectively inhibit the increase in neutrophil-derived MMP9, thus playing a protective role in the acute stage of ischemia and preventing the destruction of the BBB [312].

### 5.4. Noninvasive Brain Stimulation

Noninvasive brain stimulation (NIBS) can promote positive neuromodulation by targeting microglia (Table 4). Repetitive transcranial magnetic stimulation (rTMS) has been extensively used in stroke rehabilitation because it is non-invasive, painless, and safe. It is widely accepted that high-frequency (≥5 Hz) and intermittent theta-burst stimulation (iTBS) can improve neuronal excitability, whereas low-frequency (≤1 Hz) and continuous theta-burst stimulation (cTBS) can inhibit neuronal excitability [313]. Its mechanisms mainly include regulation of brain excitability, improvement of BBB permeability, regulation of neurotransmitters and cytokine levels, and reconstruction of brain networks [314]. In addition, rTMS can improve the nervous microenvironment and regulate microglial activation and polarization. Studies have shown that theta-burst transcranial magnetic stimulation prominently reduces the levels of pro-inflammatory cytokines and chemokines and inhibits pyroptosis-related proteins in neurons around infarctions. By inhibiting the TLR4/NF-κB/NLRP3 signaling pathway and activating STAT6, the levels of proteins associated with the M2-like phenotype are increased (IL-4, IL-10, Arg-1, and CD206) [281,283,284].

In addition, rTMS treatment has other therapeutic effects, including inhibition of glial scar formation, reduction of neuronal deformation and synaptic loss, maintenance of mitochondrial membrane integrity [281], increased expression of genes related to nerve remodeling, repair, neuroprotection, and damage responses [315], and promotion of the proliferation of neural stem/progenitor cells (NSPC) [316]. In conclusion, rTMS can improve the inflammatory microenvironment by regulating the microglial phenotype and promoting the functional recovery of the CNS.

Transcranial ultrasound stimulation (TUS) or transcranial focused ultrasound stimulation (tFUS), a new non-invasive brain stimulation method, has high penetration depth and high spatial resolution and reduces ischemic injury and neuroprotective effects after stroke [317]. It can be used in arterial thrombolytic therapy to promote the functional recovery of patients with ischemic stroke and improve the curative effect of rehabilitation [318]. tFUS can activate microglia [319] and upregulate the IL-10 signaling pathway to regulate microglial polarization towards an anti-inflammatory phenotype [292]. Other studies have shown that low-intensity transcranial pulsed ultrasound can induce the expression of BDNF, antagonize the hypoxia/reperfusion-induced microglial injury [290], and reduce the proportion of damaged neurons after stroke [320]. Therefore, timely and appropriate tFUS intervention after the onset of ischemic stroke can improve neurological function and quality of life in post-stroke patients.

Transcranial direct current stimulation (tDCS) with different polarities may play a dual role in ischemic brain. Cathodic stimulation protects cortical neurons from ischemic injury and reduces inflammation. However, the anode can aggravate impairment of the BBB, increase the exosmosis of immunoglobulin G (IgG) in circulating blood, reduce the tight connection of blood vessels, and increase the volume of injury [287]. During the acute stage of stroke, the release of cortical glutamate [287] and activation of microglia are decreased by cathodic stimulation [285]. Cathodic tDCS has been reported to accelerate functional recovery, increase neurogenesis, and reduce M1-type microglia-associated CD16/32 expression between days 5 and 9 post-stroke. During the second week, microglia became more polarized towards the neuroprotective CD206+ M2 phenotype [289]. These changes were more concentrated in the ischemic core [286].

In addition to the damaging effects, anodic tDCS has been found to have a positive effect in the subacute and chronic stages of post-stroke [287]. Anodic tDCS has the potential to modulate ischemic penumbra and contralateral pericortical dendrite and axonal plasticity without exacerbating the infarct volume and metabolic changes [321].

The mechanism of transcranial direct current stimulation for stroke treatment is not fully understood, but several studies have shown that tDCS can affect neuronal function by regulating the activation and polarization of microglia. It is also important to explore the optimal time window for tDCS application.

Photobiomodulation (PBM) has been shown to have neuroprotective or neurorepair effects in patients with chronic stroke through transcranial irradiation and multizone irradiation [322]. Red and near-infrared light can regulate microglial activity, reduce oxidative stress and inflammatory responses, and promote neurogenesis [323]. Vogel et al. have shown that transcranial low-level laser-induced photobiological regulation can inhibit microglial overactivation, enhance the expression of glial fibrillary acid protein (GFAP), and ultimately decrease neuroinflammation and infarct lesion volume [324].

Although different therapeutic interventions can target and regulate microglia in order to promote stroke recovery, they have several limitations. Drugs that enter the body orally and intranasally are unable to concentrate and act efficiently on lesions, resulting in low bioavailability. Many drugs can be injected intravenously, but it is difficult for drug molecules to cross the BBB to directly target microglia [325]. This may limit the effectiveness of the treatments. For exercise and noninvasive brain stimulation, the selection of types and control of parameters, such as the intensity, duration, frequency, may directly affect the therapeutic effect. Since research targeting microglia is still at the stage of animal studies, rodents may experience stress at a high therapeutic intensity and cause a detrimental response [280]. In cell-based therapy, although clinical studies have demonstrated the safety and efficacy of cell transplantation [326,327], an animal study showed that transplantation of iPSCs led to tumorigenesis and aggravated ischemic injury in mice [328]. Therefore, it is necessary to monitor the risk of carcinogenesis following cell transplantation. Moreover, although the effect of the therapy is robust in different study species, the clinical potential of cell-based therapies should be further explored.

## 6. Conclusions and Perspective

Microglia play an important role in maintaining homeostasis in the CNS during the resting state. During stroke recovery, microglia are activated and play both beneficial and detrimental roles, which are closely related to the polarization and phenotypes of microglia. While single-cell sequencing has provided new insights into the classification and exploration of microglial subsets, the regulation of microglia directed to the beneficial state after stroke remains to be further studied. Second, most of the molecular switches and signaling pathways are activated in the acute phase of stroke, and whether they can play the same role in the chronic phase needs to be studied further. This mechanism may provide the molecular basis for rehabilitative intervention targeting microglia after stroke. Third, the structural differences between animal models and the human brain result in different microglial distribution and activation patterns. The clinical study of rehabilitative interventions targeting microglia should also be strengthened in stroke recovery. Fourth, many studies have regarded microglia as key targets in neuroplasticity regulation for post-stroke rehabilitation. In addition to the therapeutic methods mentioned in this article, many combination therapies may achieve better efficacy, thus warranting further study. For example, in addition to its neuromodulatory effects, transcranial focused ultrasound therapy has been shown to improve blood supply and repeatedly open the BBB [329]. Therefore, drug delivery during transcranial focused ultrasound therapy allows drug molecules to readily pass through the BBB, which has multiple therapeutic effects, and provides new ideas for combining drug therapy after stroke. Fifth, although the spatiotemporal colocalization of microglial activation and the development of neuroplasticity in stroke recovery have been presented, more solid evidence that microglia directly regulate neuroplasticity after stroke, which is promising for post-stroke rehabilitation, still needs to be further accumulated.

## Figures and Tables

**Table 1:** Pathophysiology and therapeutic targets of ischemic stroke in the acute, subacute, and chronic phases. BBB, blood-brain barrier; ROS, reactive oxygen species.

	Acute Phase of Stroke	Subacute Phase of Stroke	Chronic Phase of Stroke
Time course	Minutes to days	Days to weeks	Weeks to mouths
Pathophysiological mechanisms	Infiltration of peripheral immune cells; activation of resident glial cells; disturbance of ionic homeostasis; oxidative stress; BBB destruction; mitochondrial dysfunction; DNA damage	Amplification of immune responses; increased ROS production; cell edema and ion imbalances	Decrease in excitotoxicity; protective inflammatory response
		Axonal sprouting, dendrite remodeling; neurogenesis, angiogenesis; increased levels of growth factors	
Consequences	Cell injury or death	Onset of neuroplasticity	Tissue repair
Therapeutic targets	Neuroprotection; reducing reperfusion injury	Functional rehabilitation	

**Table 2:** Single-cell profiles of specific microglia subsets at different developmental stages and pathological states. *Ctsb*, cathepsin B; *Ctsd*, cathepsin D; *Lamp1*, lysosomal associated membrane protein 1; *Apoe*, apolipoprotein E; *Tmsb4x*, thymosin beta 4 X-linked; *Eef1a1*, eukaryotic translation elongation factor 1 alpha 1; *Rpl4*, ribosomal protein L4; *Cst3*, cystatin 3; *Tmem119*, transmembrane protein 119; *Selplg*, selectin P ligand; *Slc2a5*, solute carrier family 2 member 5; *Malat1*, metastasis associated lung adenocarcinoma transcript 1; *Lgals3*, galectin-3; *Cst7*, cystatin F; *Ccl4*, C-C motif chemokine ligand 4; *Ccl3*, C-C motif chemokine ligand 3; *Il1b*, interleukin 1 beta; *Id2*, inhibitor of DNA binding 2; *Atf3*, activating transcription factor 3; *Ifitm3*, interferon induced transmembrane protein 3; *Rtp4*, receptor transporter protein 4; *Oasl2*, 2′–5′ oligoadenylate synthetase-like 2; *Lpl*, lipoprotein lipase; *P2ry12*, purinergic receptor P2Y12; *Cx3cr1*, C-X3-C motif chemokine receptor 1; *GPNMB*, glycoprotein nmb; *HSP90AA1*, heat shock protein 90 alpha family class A member 1; AD, Alzheimer’s disease; PD, Parkinson’s disease.

Species	Model	Microglia Subset	Gene	Protocol	Reference
CD-1 mice	In embryonic development	The subsets during CNS development and homeostasis in the adult brain	*Ctsb*, *Ctsd*, *Lamp1*, *Apoe*, *Tmsb4x*, *Eef1a1*, *Rpl4*, *Cst3*	SMART-seq2	[101]
	In postnatal development		*Tmem119*, *Selplg*, *Slc2a5*, *Malat1*		
C57BL/6J mice	Aging brain (P540)	Aging clusters (OA) 2	*Lgals3*, *Cst7*, *chemokines Ccl4*, *Ccl3*, *Il1b*, *Id2*, *Atf3*	10 × Genomics	[102]
		Aging clusters (OA) 3	*Ifitm3*, *Rtp4*, *Oasl2*		
Heterozygous 5XFAD transgenic mice	AD	Microglia type associated with neurodegenerative diseases (DAM)	*Apoe*, *Lpl*, *Cst7* ↑ *P2ry12*, *Cx3cr1*, *Tmem119* ↓	MARS-seq	[103]
Frozen human post-mortem midbrain tissue sections	Idiopathic PD	Microglia cluster involving inflammatory response	*IL1B*, *GPNMB*, *HSP90AA1*	10 × Genomics	[104]

## Data Availability

Not applicable.
